# Typhoid Fever and Its Association with Environmental Factors in the Dhaka Metropolitan Area of Bangladesh: A Spatial and Time-Series Approach

**DOI:** 10.1371/journal.pntd.0001998

**Published:** 2013-01-24

**Authors:** Ashraf M. Dewan, Robert Corner, Masahiro Hashizume, Emmanuel T. Ongee

**Affiliations:** 1 Department of Spatial Sciences, Curtin University Western Australia, Bentley, Western Australia, Australia; 2 Institute of Tropical Medicine, Nagasaki University, Nagasaki, Japan; University of California, San Diego School of Medicine, United States of America

## Abstract

Typhoid fever is a major cause of death worldwide with a major part of the disease burden in developing regions such as the Indian sub-continent. Bangladesh is part of this highly endemic region, yet little is known about the spatial and temporal distribution of the disease at a regional scale. This research used a Geographic Information System to explore, spatially and temporally, the prevalence of typhoid in Dhaka Metropolitan Area (DMA) of Bangladesh over the period 2005–9. This paper provides the first study of the spatio-temporal epidemiology of typhoid for this region. The aims of the study were: (i) to analyse the epidemiology of cases from 2005 to 2009; (ii) to identify spatial patterns of infection based on two spatial hypotheses; and (iii) to determine the hydro-climatological factors associated with typhoid prevalence. Case occurrences data were collected from 11 major hospitals in DMA, geocoded to census tract level, and used in a spatio-temporal analysis with a range of demographic, environmental and meteorological variables. Analyses revealed distinct seasonality as well as age and gender differences, with males and very young children being disproportionately infected. The male-female ratio of typhoid cases was found to be 1.36, and the median age of the cases was 14 years. Typhoid incidence was higher in male population than female (χ^2^ = 5.88, p<0.05). The age-specific incidence rate was highest for the 0–4 years age group (277 cases), followed by the 60+ years age group (51 cases), then there were 45 cases for 15–17 years, 37 cases for 18–34 years, 34 cases for 35–39 years and 11 cases for 10–14 years per 100,000 people. Monsoon months had the highest disease occurrences (44.62%) followed by the pre-monsoon (30.54%) and post-monsoon (24.85%) season. The Student's t test revealed that there is no significant difference on the occurrence of typhoid between urban and rural environments (p>0.05). A statistically significant inverse association was found between typhoid incidence and distance to major waterbodies. Spatial pattern analysis showed that there was a significant clustering of typhoid distribution in the study area. Moran's I was highest (0.879; p<0.01) in 2008 and lowest (0.075; p<0.05) in 2009. Incidence rates were found to form three large, multi-centred, spatial clusters with no significant difference between urban and rural rates. Temporally, typhoid incidence was seen to increase with temperature, rainfall and river level at time lags ranging from three to five weeks. For example, for a 0.1 metre rise in river levels, the number of typhoid cases increased by 4.6% (95% CI: 2.4–2.8) above the threshold of 4.0 metres (95% CI: 2.4–4.3). On the other hand, with a 1°C rise in temperature, the number of typhoid cases could increase by 14.2% (95% CI: 4.4–25.0).

## Author Summary

This research studies the spatial and temporal distribution of typhoid infections in the Dhaka metropolitan area of Bangladesh in the period 2005 to 2009. Data from hospital admission records was analysed together with a range of demographic, environmental and climatic data, in what is believed to be the first study of this nature; clear periodicity was found in the timing of case occurrences, with most cases occurring in the monsoon season. Men and very young children appear to be at greatest risk of contracting the disease. Closeness to rivers was also found to be a contributor to increased typhoid risk. While a difference in rates between urban and rural locations suggested by other studies was not found, distinct clustering of the disease was uncovered. Two of these clusters are located in central Dhaka with a third in the north of the metropolitan area.

## Introduction

Typhoid fever is one of the leading causes of morbidity and mortality across the world [1].Typhoid is caused by a bacterium of the genus *Salmonella*. Salmonella infection in humans can be categorised into two broad types, that caused by low virulence serotypes of *Salmonella enterica* which cause food poisoning, and that caused by the high virulence serotypes *Salmonella enterica typh*i (*S. typhi*), that causes typhoid,and a group of serovars, known as *S Paratyphi* A, B and C, which cause Paratyphoid [2]. Humans are the only host of this latter group of pathogens. *S. Typhi* is a highly adapted human-specific pathogen [3], and the illness caused by these bacteria is a serious public health concern, particularly in developing countries [4]. A recent estimate found that 22 million new typhoid cases occur each year in the world with some 200,000 of these resulting in death [5], indicating that the global burden of this disease has increased steadily from a previous estimate of 16 million [6] however, case-fatality rates have decreased markedly [5]. The highest number of cases (>100 per 100,000 persons/year) and consequent fatalities are believed to occur in South Central and Southeast Asia [1]. Generally, typhoid is endemic in impoverished areas of the world where the provision of safe drinking water and sanitation is inadequate and the quality of life is poor.

Although contaminated food [7]–[11] and water [9], [12]–[15] have been identified as the major risk factors for typhoid prevalence, a range of other factors have been reported in different endemic settings such as poor sanitation [16], close contact with typhoid cases or carriers [17], level of education, larger household size, closer location to water bodies [17], [18], flooding [19], personal hygiene [12], poor life style [20], and travelling to endemic areas [21]. In addition, climatic variables such as, rainfall, vapour pressure and temperature have an important effect on the transmission and distribution of typhoid infections in human populations [12], [22].

On the Indian subcontinent, Pakistan has the highest incidence (451.7 per 100,000 persons/year) of typhoid fever followed by India (214.2 per 100,000 persons/year) [23]. The mean age of those infected with typhoid is 15.5 years in India and 7.0 years in Pakistan. Bangladesh, located in South Asia, has a population that is mostly impoverished; thus, it is probable that typhoid incidence will be high. A population-based study reported that children and young adults had the highest age-specific rates of all enteric infection [24]. Typhoid disproportionately affects children, with the highest incidence rate being observed in children <5 years old [25].Their study also indicated distinct seasonal patterns in the occurrence of typhoid. A community-based study in an urban slum in Bangladesh, by Brook et al. [26] suggested that the overall incidence was 3.9/1000 persons/year and the rate was higher in preschool children aged between 0 and 4 years (18.7 per 1000 persons/years). A recent study revealed that typhoid fever was endemic in urban areas with a high-incidence of multi-drug resistant strains [4]. This study also found that the incidence rate is higher in children aged <5 years (10.5/1000 persons/year) with an overall incidence rate of 2.0/1000 persons/year. Age-wise data demonstrated that the infection was lower in an older group (0.9/1000 persons-year) than in children [4]. Both of these studies [4], [26]) reported that the incidence of typhoid fever among populations in the urban slums of Dhaka was higher than that of paratyphoid. In a group of low income children in a semi-urban setting, the prevalence of typhoid was also found to be high in school age children [27].

Although Bangladesh is located in a region in which typhoid is highly endemic [5], very little is known about the spatial and temporal distribution of typhoid on a regional scale. Population-based studies in Dhaka have highlighted the high disease burden but did not address the spatial distribution of the population at risk from this disease; therefore, they cannot be used to make generalizations for the entire metropolitan population [20].

Timely data are essential not only to introduce vaccination programs for typhoid [23] but also to identify populations at risk so that public health interventions can be carried out effectively. A better understanding of the spatial distribution of diseases such as typhoid, together with the associated environmental factors can support more targeted disease control efforts. In addition, a spatio-temporal analysis can assist in understanding the disease dynamics in regions where the infection is already a concern.

Geographic Information Systems (GIS) are an important tool in understanding the distribution of diseases over space, and such systems have contributed significantly to spatial epidemiological research [28], [29]. GIS enables the factors associated with a disease can be investigated spatially, thereby allowing researchers to test different hypotheses. This is achieved by incorporating geocoded data from a wide range of sources such as census, surveys and remote sensing satellites [30]. As a GIS is capable of analysing disease data using a range of spatial analytical tools and spatial statistics, the output can provide valuable information in the study of public health issues enabling health officials to plan for informed decision making [29], [31]. GIS and spatial statistics have previously been applied to identify spatial clustering, risk areas and causative factors in historical studies of *S. typhi* in the USA [32] and India [18].

We aimed to study typhoid infection in the Dhaka Metropolitan Area (hereinafter, DMA) of Bangladesh with the following specific goals: (i) to analyse the epidemiology of typhoid cases from 2005 to 2009; (ii) to identify spatial patterns of typhoid infection based on two spatial hypotheses; and (iii) to determine the hydro-climatological factors associated with the prevalence of typhoid.

The spatial hypotheses in ii) above are: a), the infection rate of typhoid is higher among people living close to water bodies, and b) there is no significant spatial clustering of the disease in the study area.

## Materials and Methods

### Study area

**Figure 1:**
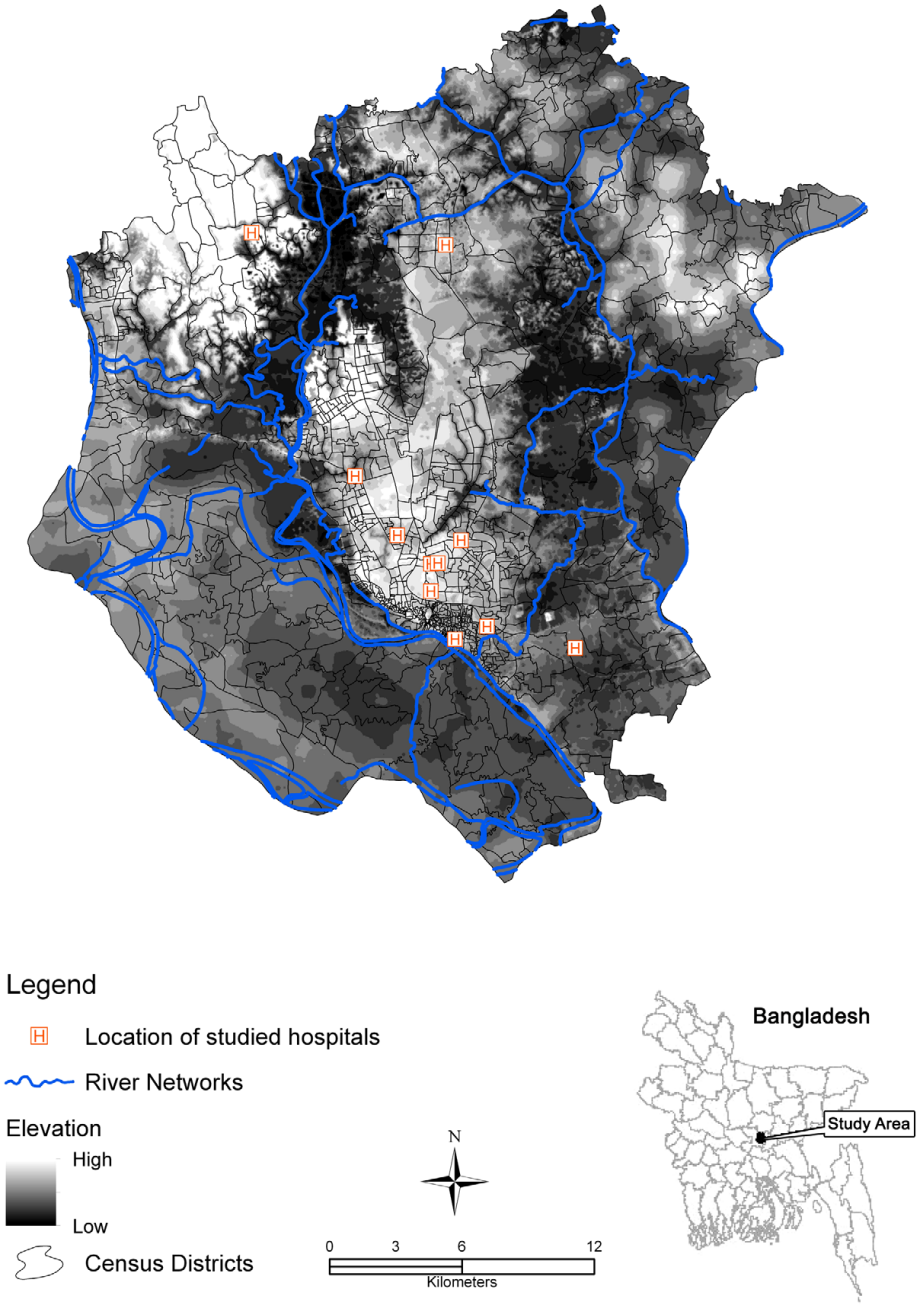
The Dhaka Metropolitan Area (DMA).

The study area of DMA is that area contained within the Dhaka Metropolitan Development Plan (DMDP). The DMA comprises three municipalities, Dhaka City Corporation (DCC), the municipalities of Savar and Tongi, and numerous other local government areas (*unions*). The area is located between 23.61° and 23.97°N and 90.22° and 90.59°E, and has an area of 878 km^2^ (Figure 1). Based on the most recent published census (2001), the total population of this area was more than 8 million with an average literacy rate of 65% [33]. Topographically, the area is flat with a surface elevation ranging from 1 to 16 meters. The study area is surrounded by five major river systems, namely the Buriganga, Turag, Tongi, Lakhya and the Balu rivers, which flow to the south, west, north, east and northeast, respectively. These rivers are primarily fed by local rainfall but some are also distributaries of the considerably larger Ganges, Brahmaputra and Meghna rivers. The city has a humid sub-tropical monsoon climate and receives approximately 2000 mm of rainfall annually, more than 80% of which falls during the monsoon season from July to October. Most of the inhabitants in the three municipal areas have access to piped-water but outside the municipalities, drinking water sources may include ponds, rivers, tube wells etc.

### Typhoid case data

Data on typhoid case occurrence for the period 2005 to 2009 was collected from the record rooms of each of the 11 major hospitals located in the study area (Figure 1) during the period from April to December 2009. The 30-member data collection team included medical graduates, information technologists and geographers. The team members were trained for two days by various professionals including medical practitioners, epidemiologists, information technologists and environmentalists. Four of the team members were responsible for the overall supervision of data collection and four were responsible for data encoding. A standardized abstraction form was created after consultation with epidemiologists, medical practitioners and environmentalists. The form was designed to document the patient's residence, demographic and clinical data, date of admission, date of discharge and outcome (alive/dead). Only those admitted to hospital with typhoid fever were included in the database, and outpatients were not included. Outpatient records in the study area are not systematic enough to enable their inclusion in this study and may include patients with other less serious febrile and diarrheic conditions. The diagnosis of typhoid was made by physicians at the respective hospitals using either blood culture or Widal tests [3]. To avoid data duplication, we first matched data using all the demographic variables and then cross-checked the data against the corresponding day/year in the log books of the hospitals. If a case satisfies both of these records, then we included in the database. We excluded cases residing outside of DMA along with the duplicates (n = 1231). This resulted in a total of 4355 cases pertaining to the study area. To minimize error in case mapping, we also cross-referenced individual case's place of residence with the 2001 census tract names by Bangladesh Bureau of Statistics (BBS). When place of residence inconsistencies were found, we used the smallest suitable mapping unit since people in the study area are more familiar with local names than with administrative units. In practice these units were the *mahalla* the *mauza*. A *mahalla* is the smallest urban administrative geographic unit and has a Ward Council Institution. It is a settlement of homogenous group of people of urban municipal areas. A *mauza* is the smallest rural geographic unit having Jurisdiction List Number (JLN). It has a definite area demarcated by the revenue department and refers exclusively to rural areas.

### Population and geographic data

A spatio-temporal database of the census tracts in the study area was created from the small area atlas of BBS, digital databases from Bangladesh Space Research and Remote Sensing Organization (SPARRSO) and the Centre for Environment and Geographic Information Systems (CEGIS) database. Whilst this database was being created, it was found that 25 new census tracts used in the 2001 census, were not identified in the existing digital spatial data. To identify these, the 1991 census tracts names were first matched with the 2001 census tracts names using the community series of BBS. A hard copy map from BBS, which highlighted the road networks that were used to split the original (1991) census tracts to create new census tracts for 2001 census, was used to digitise the tracts created between decennial censuses. Field visits using a high resolution mobile mapping GPS (Trimble Nomad 800GXE) were used to confirm and correct the road network locations. The final census tract layer consists of 1212 polygons which include 441 rural villages (*mauza*) and 771 urban communities (*mahallas*). All typhoid cases were then aggregated and matched with the census tract polygon features using ArcGIS 10 software since downscaling spatial units into census districts has been shown to provide valuable information on the spatial distribution of disease [33]. Data for basic geographic features such as major waterbodies were obtained from the CEGIS.

The population data were acquired from the BBS community series [34], encoded in a spreadsheet and then linked with the census tract boundary using a unique ID. The population data between 2005 and 2009 were interpolated assuming a uniform rate of population growth during the decade in each census tract. The following equation was used to estimate the population for the years 2005 to 2009:
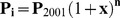
(1)Where, P_i_ is population for year of interest, P_2001_ is the population of 2001, x is the annual growth rate between 1991 and 2001, and n is the number of years between 2001 and the year of interest.

The interpolated population data were used as the denominators to calculate the typhoid incidence rate for each year. We have not attempted to interpolate other demographic variables such as age, sex and age-wise population, hence the relevant fractions from the 2001 census were used to calculate the age-specific incidence rate for each age group reporting typhoid fever.

### Environmental data

Daily river level data for four rivers (the Buriganga, Tongi, Turag and Balu) across the DMA were acquired from Bangladesh Water Development Board (BWDB). The daily average of the maximum water levels at the four stations was used in the analysis. In addition, climatic data including daily rainfall, temperature and humidity were obtained from Bangladesh Meteorological Department. The mean daily temperature and total weekly rainfall were calculated from this daily climate database.

### Descriptive statistics

The typhoid cases and demographic data were used to examine typhoid epidemiology in DMA. The annual incidence rate for each census district for each year was calculated as:
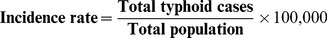
(2)To investigate the monthly and seasonal variations, we have categorised annual disease data into three distinct seasons, i.e. pre-monsoon (March to June), monsoon (July–October) and post-monsoon (November to February). To determine the most vulnerable age group to typhoid, we estimated age-specific incidence rate per 100,000 persons. Geographic variation of typhoid occurrences in terms of urban and rural areas was determined by classifying our census tracts into two geographic entities (e.g. urban and rural) defined by BBS [35] (2001)^1^. The difference between the means for urban and rural cases was tested for statistical significance using the t-test. Temporal patterns of typhoid cases were also investigated and an epidemic curve was prepared based on the annual incidence of typhoid divided by total population for each year multiplied by 100,000 persons.

### Spatial analysis and modeling spatial dependency

A nonparametric statistical analysis was first performed to verify the spatial association between geographic data and typhoid incidence rate. We assumed that people living closer to water bodies (e.g. rivers) had a higher rate of typhoid infection than people living further away. A surface representing distance from waterbodies was generated and the distance from the centroid of each census district from the water bodies was then measured. This resulted in a table of, typhoid incidence rate and distance from water bodies for each census district. The incidence rate was categorised into five classes: 0–5 cases; 5–10 cases; 10–15 cases; 15–20 cases and 20 + cases per 100,000 persons. Likewise, the distances were classified as: 0–1 km; 1–2 km; 2–3 km; 3–4 km and 4 km +. Finally, a contingency table was created and a nonparametric chi-square (χ^2^) test was performed.

In order to ascertain the results of noparametric chi-square (χ^2^), spatial regression in terms of Geographically Weighted Regression (GWR) was performed. In carrying out the GWR modelling, the data was subset to include only study units (*mauza* and *mahalla*) that registered at least one typhoid case over the study period. This was done to ensure that all areas participating in the analysis were coming from within the catchment areas of the 11 major hospitals from which the disease data was collected. It was assumed that areas that recorded zero incidences of typhoid infection over the five year periods never sought medical attention from these hospitals.

First of all, an Ordinary Least Square (OLS) regression was performed using incidence (log-transformed) data as dependent variable against distance to major waterbodies as independent variable. This resulted in the following statistics (R^2^ = 0.052, coefficient = −0.00031, p<.05), AICc = 2520.925). Following the OLS, we computed spatial weights using the Queen's case contiguity rule (first order) and used for deriving Moran's *I* statistics to account for spatial autocorrelation with the following outcome (I = 0.151, p<0.05), suggesting the presence of spatial dependency in the data, which explains the poor correlation result found by OLS. Next, GWR was carried out to investigate the dependency and highlight local variability. Since the spatial configuration of features being analysed were inhomogeneous [36], we used adaptive kernel to solve regression analysis. In order to understand the model fit and compare the results of global model (e.g. OLS) with local model (e.g. GWR) [37], the Akaike Information Criterion (AICc) was used to modulate the bandwidth. Local collinearity, independency and normality of residuals of GWR were further evaluated by condition number and achieving a largest condition number of 17, much smaller than a typical value of 30, revealed that our model was free from statistical concerns. The regression diagnostics from GWR is as follows (R^2^ = 0.475, AICc = 2386.95). The spatial regression was carried out using ArcGIS (v. 10) and the R software (SPGWR Package).

### Cluster mapping

The second hypothesis of our study was that there is no association in typhoid occurrence among neighbouring spatial units. The alternative hypothesis was that neighbouring locations have similar typhoid rates: in other words, spatial clustering exists.

Since the census tracts considered in this study are highly variable in terms of shape, size and population distribution, use of raw incidence rate may not fully represent the relative magnitude of underlying risk since variation in population size between census districts means that standardised mortality/morbidity ratios (SMR) imprecisely estimated, from only a few cases, may dominate the spatial pattern. To overcome this problem, we have applied the Empirical Bayesian (EB) smoothing technique [38] to our typhoid data. This method adjusts raw rates by incorporating data from other neighbouring spatial units [39]. Essentially, the raw rates get ‘shrunk’ towards an overall mean which is an inverse function of the variance. Application of EB smoothed incidence rate not only provides better visualization compared to unsmoothed rate but also serves to find true outliers [40]. The next step was to conceptualise the spatial relationship that defines neighbourhood structure around each census district. We defined the neighbourhood structure of each census tract as being first order Queen's Case. Global Moran's *I* was used to initially evaluate spatial clustering. If spatial autocorrelation was found, we then evaluated the location of typhoid clusters using local indictors of spatial autocorrelation (LISA) [41]. Inference for significance of both global Moran's *I* and LISA was based on 999 Monte Carlo randomizations using the GeoDa software [42]. An alpha level of 0.01 was set to test the statistical significance. Based on these permutations and threshold, we have plotted values on a map to display the location of typhoid clusters in DMA.

### Time series relationship between typhoid cases and hydro-climatological factors

We examined the relationship between the weekly number of typhoid cases and river levels and weather variables (temperature and rainfall) using generalised linear Poisson regression models allowing for overdispersion [43]. To account for the seasonality of typhoid counts not directly related to the river levels and weather, Fourier terms up to the 8th harmonic were introduced into the model. Fourier terms can capture repeated periodic (e.g. seasonal) patterns comprising a combination of pairs of sine and cosine terms (harmonics) of varying wavelengths [44]. This number of harmonics was chosen as that which minimised the Akaike Information Criteria. Additional indicator variables for the years of the study were incorporated into the model to allow for long-term trends and other variations between years. In this case an indicator variable for public holidays was incorporated into the model to control bias in the event that holidays affected access to hospital. To allow for the temporal autocorrelations an autoregressive term of order 1 was also incorporated into the model [45].

To account for delays in the effect of river levels and weather variables on the number of typhoid cases, temporally lagged river level and weather variables were incorporated into the model. To identify the optimum lag period, linear and spline terms for average river levels and weather variables up to each lag were incorporated into a model comprising indicator variables of years and public holidays and Fourier terms. The smallest lag with a statistically significant association (p<0.05 by Wald test) was chosen as the optimal lag. The optimal lag for river level effect was from 0 to 5 weeks (the average of the river levels in a given week and in the 5 previous weeks), and the optimal lags for rainfall and temperature effect were found to be 0 to 3 and 0 to 4 weeks, respectively.

The initial analysis was designed to identify the broad shape of any association. We fitted natural cubic splines (3 df) [46] to the average river level over lags 0–5 weeks, to the average rainfall over lags 0–3 weeks and to the average temperature over lags 0–4 weeks, as separate splines simultaneously included in the model. We included all the variables in the models to control potential mutual confounding. The model took the following form:
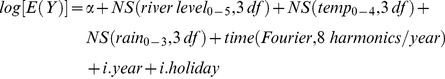
(3)where, *E(Y)* is the expected weekly case count, “*river level*”, “*temp*” and “*rain*” indicate the average weekly river level, temperature and rainfall in each lag. *NS* indicates a natural cubic spline function. *Fourier* represents the Fourier (trigonometric) terms. *i.year* represents indicator variables for the year. *i.holiday* represents an indicator variable for weeks that include public holidays.

Because the initial analysis suggested a log-linear association through the whole range of river levels and temperature, we fitted simple linear models for river levels and temperature. A log-linear association above and below a threshold was suggested for river levels and rainfall, respectively, and so we fitted a linear threshold model for river levels and rainfall. The thresholds were chosen based on the maximum likelihood estimation for the river levels and rainfall over a grid of all possible one decimal and integer values within the range indicated on the river levels and rainfall-typhoid graphs, respectively. Likelihood profile confidence intervals (CIs) for the threshold were calculated as the thresholds for which deviance of the model was 3.84 more than the minimum. An increase in the number of cases that were associated with a 0.1 metre, 10 mm and 1°C increases in a given measure of river levels, rainfall and temperature, respectively, estimated as coefficients from the regression model, were reported as percentage change.

To investigate whether or not the main results were sensitive to the levels of control for seasonal patterns, the analyses were repeated using Fourier terms of weeks up to the 10th harmonic per year adding one harmonic at a time (0 to 10 pairs of harmonics). Indicator variables of the month were also examined instead of Fourier terms.

## Results

### Descriptive analysis

**Figure 2:**
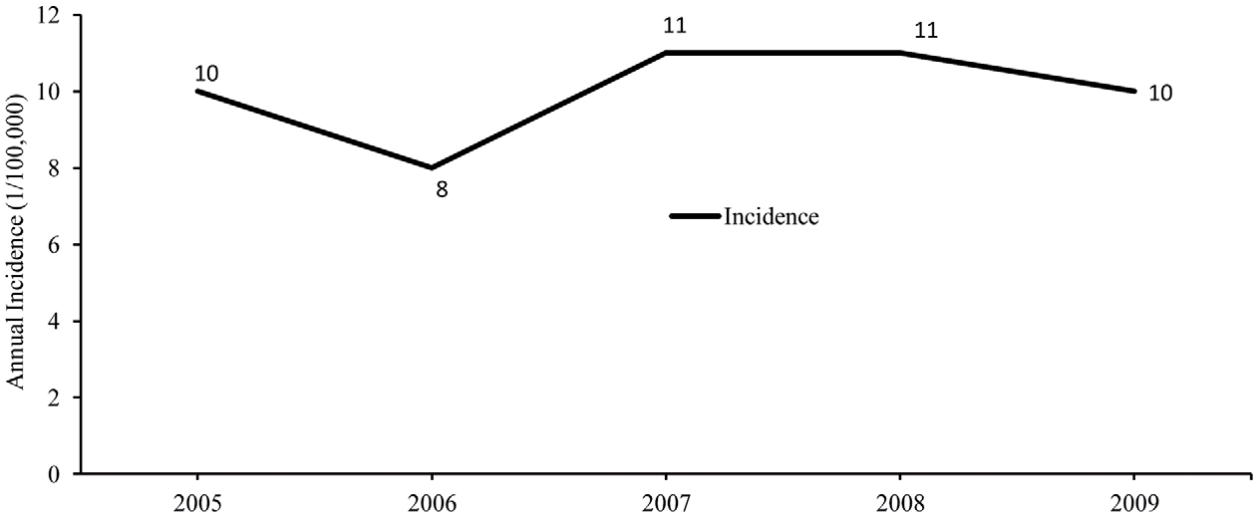
Annual typhoid incidence.

**Figure 3:**
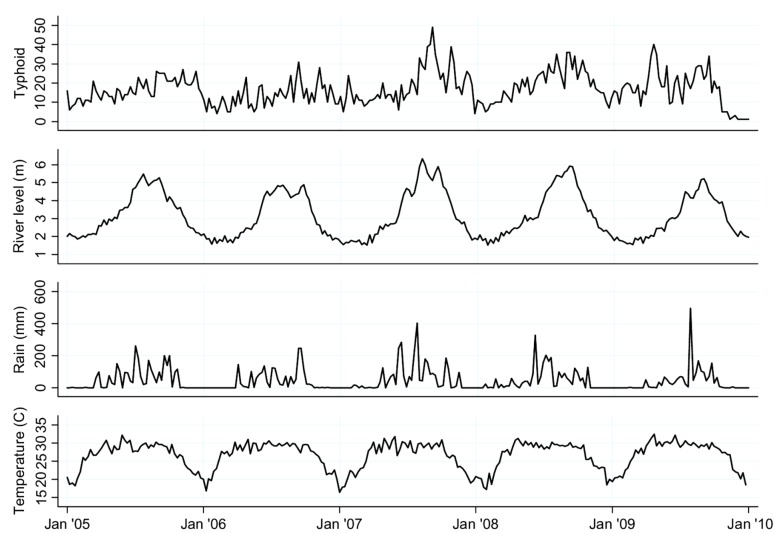
Seasonal variations in Typhoid cases. The number of Typhoid cases per week, with reference to river level, temperature and rainfall data in Dhaka Metropolitan Area, 2005–2009.

**Figure 4:**
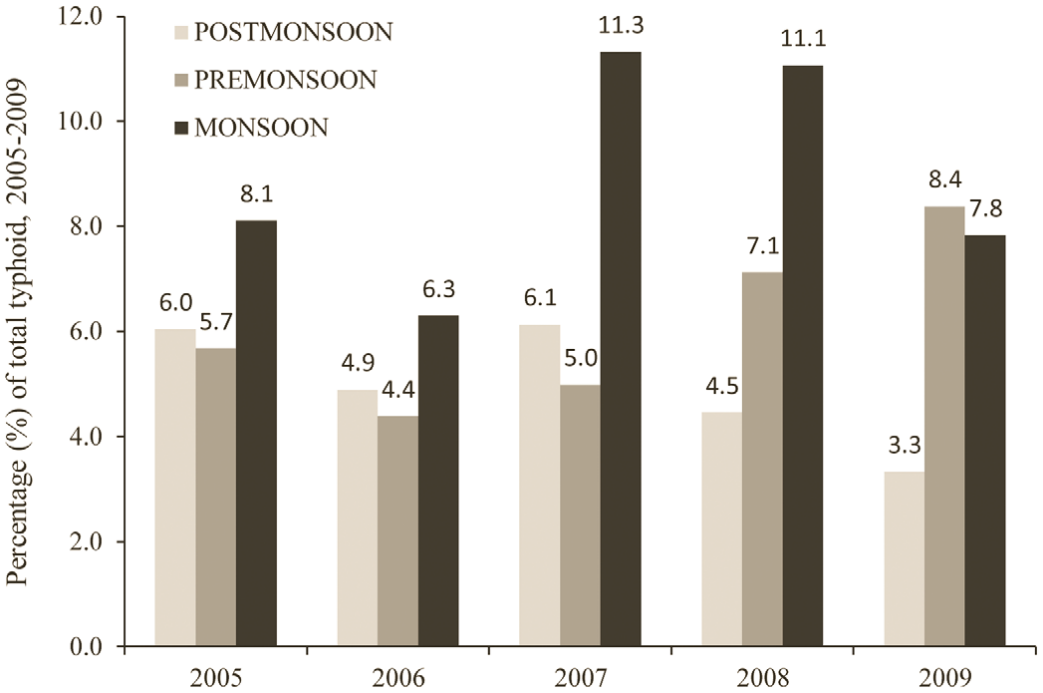
Bar graph showing percentage of seasonal typhoid cases for 2005–2009.

The annual typhoid incidence is shown in Figure 2, with the average number of typhoid cases in DMA being 871/year. Figure 3 shows the number of typhoid cases per week, and river level, temperature and rainfall data in the DMA. July–October had the highest typhoid occurrences followed by April–June (Fig. 3), and most typhoid cases were reported in monsoon season (44.62%) followed by the pre-monsoon (30.54%) and post-monsoon (24.85%) season (Figure 4).

The male-female ratio of typhoid cases was found to be 1.36, suggesting that in this population males are either more susceptible to typhoid, or more likely to present for hospital treatment, than females. The age-wise incidence rate showed a very interesting pattern because surprisingly, no cases were reported for the 5–9 years age group during our study period. The age-specific incidence rate was highest for the 0–4 years age group (277 cases), followed by the 60+ years age group (51 cases), then there were 45 cases for 15–17 years, 37 cases for 18–34 years, 34 cases for 35–39 years and 11 cases for 10–14 years per 100,000 person (Table 1). The median age of the typhoid cases was 14 years. Out of the 4,355 typhoid cases reported during the study period, 35 people died. The majority of these deaths were in patient's 35–59 years (37%) followed by 18–34 years (26%) and 60+ (14%). The highest fatality rate occurred in males (58%).

**Table 1:** Epidemiological characteristic of reported typhoid cases in DMA, 2005–2009.

Variable					
Sex		Cases (%)	Population	Annual Incidence rate (per 100,000)	Annual Incidence rate for both sex
	Male	2511 (57.66)	4548189	55	
	Female	1844 (42.34)	3597393	51	
Age					
	0–4	Male	401545	292	277.14
		Female	359439	260	
	5–9	Male	400127	0	0.00
		Female	363890	0	
	10–14	Male	482972	14	10.73
		Female	458050	6	
	15–17	Male	279568	49	45.28
		Female	246056	40	
	18–34	Male	1752085	41	37.38
		Female	1428841	32	
	35–59	Male	1048331	29	33.55
		Female	614674	39	
	60+	Male	183561	50	51.61
		Female	126443	53	

### Spatial association

Table 2 shows the result of the χ^2^ test of the relationship between distance to water bodies and the number of typhoid cases per 100,000 persons. The association was found to be significant (p<0.05). Hence, the null hypothesis that there was no association between distance to water bodies and typhoid incidence rate was rejected. Since χ^2^ does not indicate the strength and direction of the association, Kendall's τ-B and Goodman-Kruskal γ tests were used (Table 3). Both statistics revealed a negative association between typhoid incidences and distance to water bodies, demonstrating that people living closer to water bodies had a higher infection rate than people living farther away.

**Table 2:** χ^2^ values for the correlation between distances to water bodies and the Typhoid incidence per 100,000 persons.

Test statistics	Value	Degrees of freedom	Significance value
Pearson χ^2^	656.842	16	<0.001
Likelihood ratio χ^2^	547.219	16	<0.001

**Table 3:** Strength of association between distances to water bodies and the Typhoid incidence per 100,000 persons.

Coefficient	Value	Asymptotic standard error
Kendall's τ-B	−.077	0.014
Goodman-Kruskal γ	−.106	0.019

The comparison of global and local regression models indicated that GWR outperformed OLS model in terms of AICc and coefficient of determination (R^2^). The AICc value from the OLS model for the independent variable was 2520.925. In contrast, AICc value by GWR was 2386.95. The multiple R^2^ (the coefficient of determination), also showed tremendous improvement. For example, OLS derived R^2^ was 0.052 which increased to 0.475 by GWR, demonstrating a substantial improvement in the model fit to the data. Since AICc is an effective way of comparing two models [47], a difference of three points implied an important improvement to the model fit [37].

**Figure 5:**
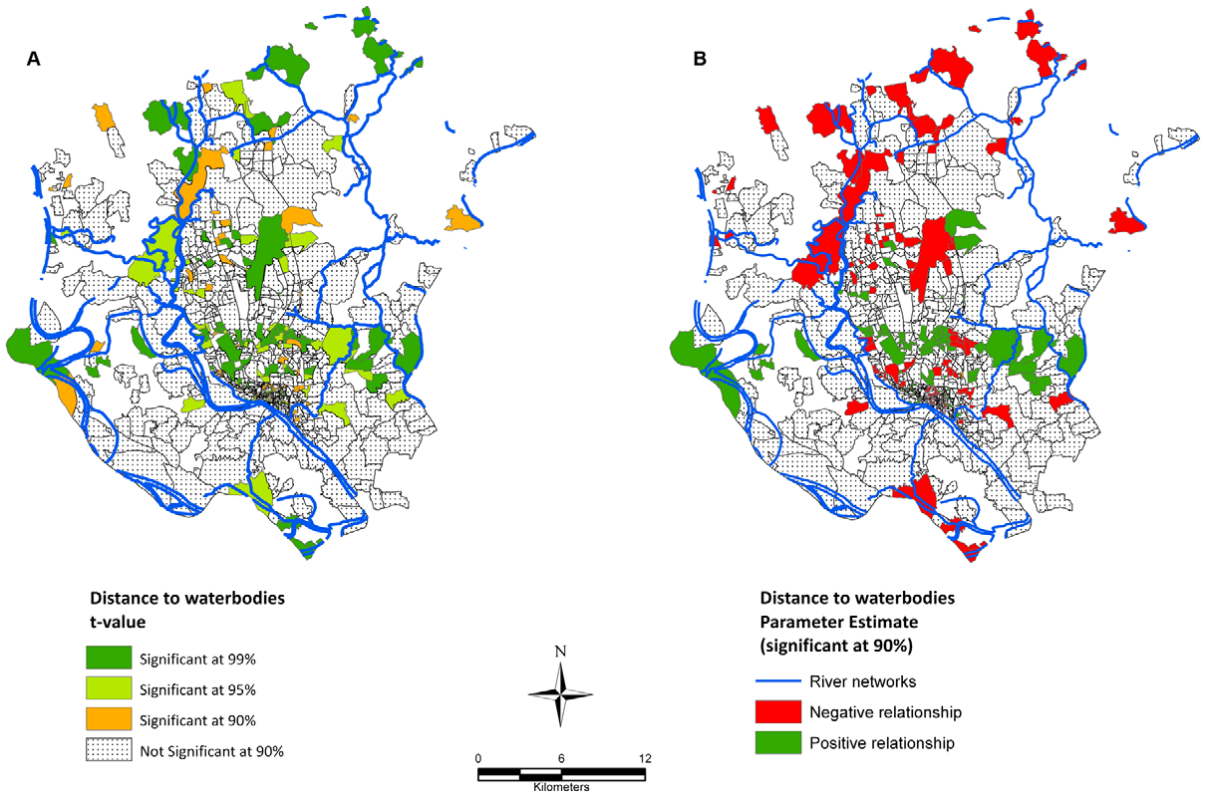
Spatial regression between typhoid incidence (per 100,000 people) and distance to water bodies. A) Shows spatial distribution of the t-value, B) shows the parameter estimates. See Figure S1 for higher resolution version.

Spatial regression analysis corroborates that spatial location was a factor in the distribution of typhoid incidence across the study area, meaning that typhoid incidence rates were associated with distance to major waterbodies (Figure 5 a&b). However, the direction of this association was two-fold with negative association being the dominant one (115 out of 197 significant observations (*mahalla* & *mauza*) showed negative relationship - 40% more than the positively associated ones, Fig. 5b). Figure 4b only shows parameter estimates (coefficients) that are significant at 90% confidence. The areas shown on the map (Fig. 5b) with significant coefficients represent a total population of 1.8 million and all together accounts for 24% of all typhoid cases (1027 out of 4355 cases), signifying the burden of disease.

### Spatial pattern analysis

**Figure 6:**
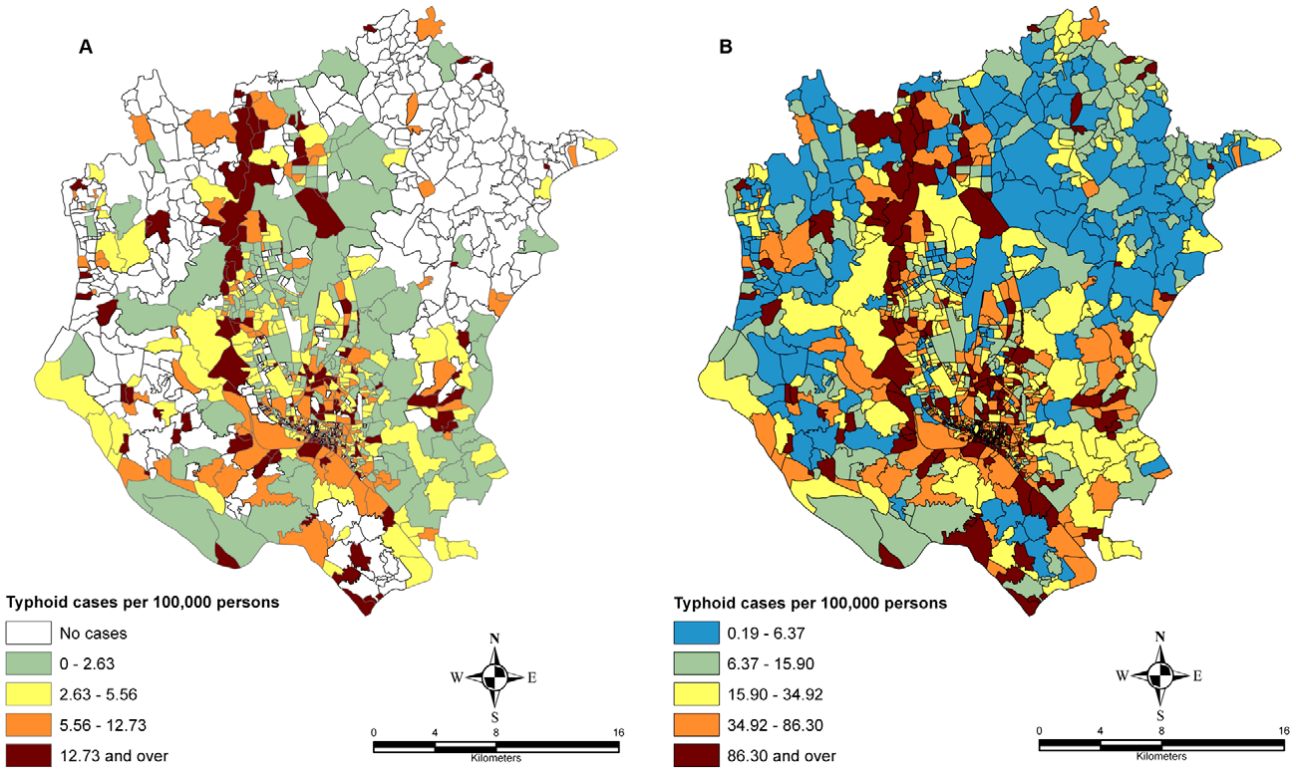
Spatial variation in the occurrence of typhoid infection. This shows the Raw annual incidence rate(A) and EB-smoothed incidence rates (B) from 2005 to 2009 in census districts in DMA: See Figure S2 for high resolution version.

The spatial pattern of the disease can be recognized easily from the smoothed and unsmoothed incidence maps (Figure 6). Geographically, the highest incidence rate was observed in the central and southern parts of DMA where the population density was relatively high. The global spatial autocorrelation analysis using the Moran's *I* is presented in Table 4. The result revealed that there was a significant clustering of typhoid distribution in DMA for each year. The Moran's *I* was highest (0.879) in 2008 (p = 0.001) and lowest (0.075) in 2009 (p = 0.0004).

**Table 4:** Global spatial autocorrelation analysis of EB-smoothed typhoid cases in DMA.

Year	EB-smoothed typhoid incidence rate		Pattern
	Moran's I	Z-Score	
2005	0.173	10.264	Clustered
2006	0.189	11.305	Clustered
2007	0.080	4.910	Weakly dispersed
2008	0.879	5.460	Clustered
2009	0.075	4.591	Dispersed

### Spatial clustering of cases

Analysis of the distribution of typhoid cases during 2005–2009 in DMA showed that typhoid was not randomly distributed. A local indictor of spatial autocorrelation (LISA) map provides the most useful information in the form of significant locations of spatial autocorrelation. While high-high and low-low locations are typically known as hot and cold spots of an event, high-low and low-high are referred to as spatial outliers [39]. The LISA map of 2005–2009 (Figure 6) revealed that there were three large multi-centred typhoid clusters and five single-centred clusters. The first multi-centred cluster was located in the old town, and was centred on 13 locations, namely Babu Bazar, A.C. Roy Road, D.C. Roy Road, Dhaka Steamerghat, Islampur, Nawabbari, Jindabazar 3^rd^ Lane, Kazi Ziauddin Road, Kumartuly, Islampur Road, Shakhari Bazar, Nalgola and Zinjira (see inset in Fig. 6). These areas are shown in the inset map in Figure 6. EB smoothed rates for these locations ranged from 96 to 2754 per 100,000 people (mean (SD): 572 (752)). The second multi-centred cluster is also located in the older part of Dhaka and comprised 7 census districts, namely Jail Road, Chak Circular Road, Purba Begum Bazar, Paschim Begum Bazar, Nazimuddin Road, Uttar Makim Katara and Ruihatta (also shown inset in Figure 6). EB-smoothed typhoid rates for these locations varied from 96 to 781 cases per 100,000 persons ((mean (SD): 246 (245)). The third multi-centred cluster is located in the northern part of DMA, also comprising 7 census tracts, including Diabari and Ashutia of Harirampur Union, Bhadam, Chak Badam, Jamaldia, Kakil Sataish and Gusulia of Tongi Municipality (see inset in Fig. 6). EB smoothed rates for these locations ranged from 283 to 1658 cases per 100,000 persons (mean (SD): 744 (559)). The single-centred locations were distributed throughout the DMA. They are Kaliganj, Gaider Tek, Chairman Bari, Bharalia and Malancha. EB-smoothed rates for these locations were 2898, 381, 904, 914 and 252 cases per 100,000 persons, respectively.

### Association of the number of typhoid cases with environmental variables

**Figure 7:**
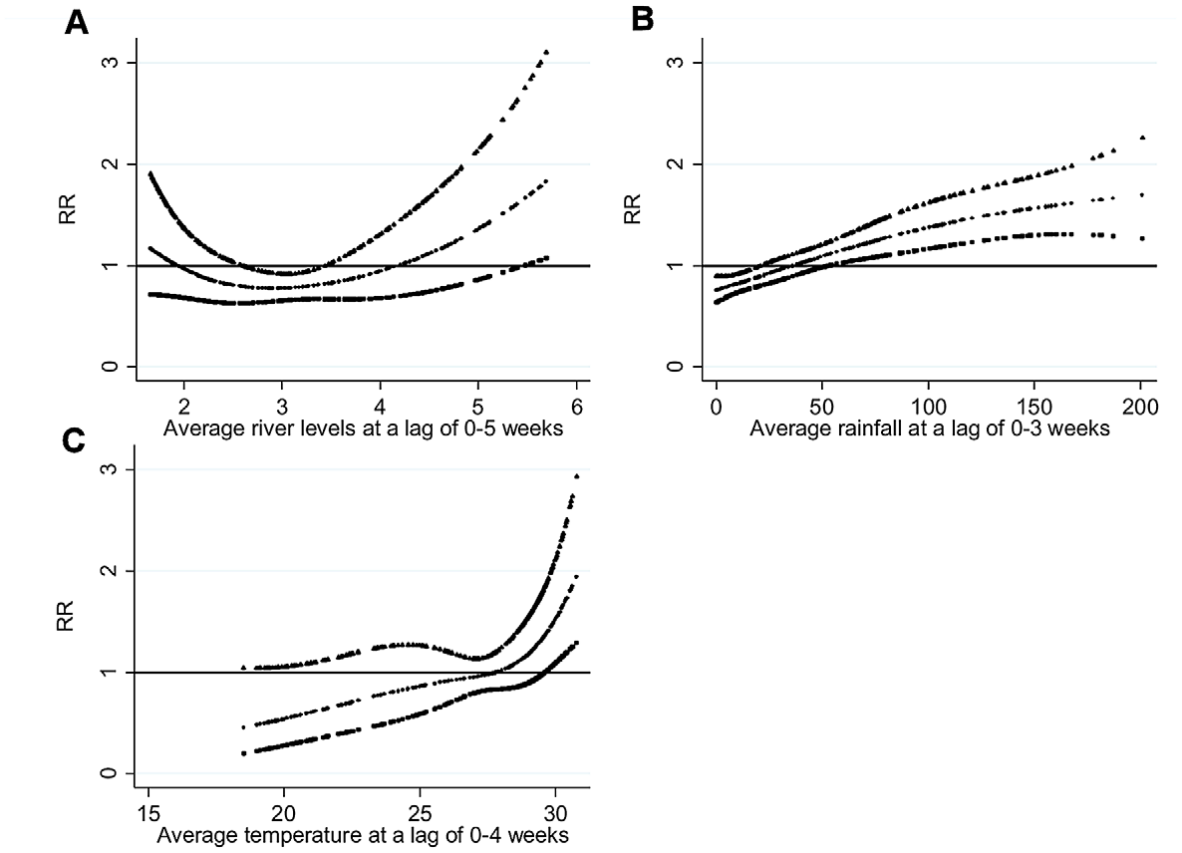
Temporal relationship between the number of typhoid cases and environmental variables. (A) Average river levels over lags of 0–5 weeks, (B) Rainfall over lags of 0–3 weeks and (C) Temperature over lags of 0–4 weeks (shown as a 3 df natural cubic spline) adjusted for seasonal variation, inter-annual variations and public holidays. RR represents the relative risk of typhoid (scaled against the mean weekly number of cases). The centre line in each graph shows the estimated spline curve, and the upper and lower lines represent the 95% confidence limits.

The relationships between the number of typhoid cases and river levels, rainfall and temperature, adjusted for season, inter-annual variations and holidays, are shown in Figure 7. An increase in typhoid cases was seen with increase in temperature, rainfall and river levels at lags of 0–4, 0–3 and 0–5 weeks, respectively. For a 0.1 metre increase in river levels, the number of typhoid cases increased by 4.6% (95% CI: 2.4–6.8) above the threshold of 4.0 meters (95% CI: 2.4–4.3). Maximum likelihood estimates of the threshold for rainfall calculated using a linear-threshold model coincided at 77 millimetres (95% CI: 54–90) for average rainfall over lags of 0–3 weeks. For a 1°C increase in temperature, the number of typhoid cases increased by 14.2% (95% CI: 4.4–25.0).

The chosen time lags of 3, 4 and 5 weeks for rainfall, temperature and river levels, respectively, are similar to those of previous studies [48], [49] although causal pathogens and study areas were different. The lag time from exposure to weather factors and access to hospitals may differ between causal pathogens. It may also differ between cities, countries and areas for topographical and population behaviour reasons.

In the sensitivity analyses, when the degree of seasonal control in the model was varied from 3 to 10 harmonics, the estimates of the effect of high river levels hardly changed, while the estimates decreased when no seasonal control was introduced or when the degree of seasonal control was 1 and 2 harmonics was introduced in the models (Figure S4).

## Discussion

To our knowledge, this is the first typhoid incidence study for this region that plots typhoid data over a five-year period together with a GIS to characterize its epidemiology and spatial patterns. Even though spatial tools have been available for quite some time, in Bangladesh, these tools have been applied mostly to diarrhoea, cholera, dengue risk, malaria and avian influenza mapping [50]–[56]. Although these studies have shown great potential in spatially identifying risk areas and associated causal factors for a range of diseases, no study has been found in the literature that applied spatial analytical tools to the geographical distribution of typhoid infections. Hence, this spatial epidemiological study of typhoid fever will be a valuable contribution to the efforts aimed at typhoid control and vaccination and prevention systems.

In common with previous findings [4], [25], we found that the age group of 0–4 had the highest incidence of typhoid. This confirms that that the rate of infection is highest in young children suggest that the current vaccination policy (which targets older age group children [25]) may need to be reassessed. Interestingly, we noticed that typhoid was prevalent in all age groups except 5–9 children group, and disproportionately affects the male population. The number of male cases surpassed female cases in the age groups of 0–4, 10–14, 15–17 and 18–34 with the highest incidence observed between the age groups of 0–4, 15–17 and 18–34. In contrast, female cases were higher in the age groups of 35–39 and 60+. Similar findings are also reported in Bangladesh [57] and in other endemic settings [16], [17], [58]. This gender preponderance might be the reflection of health-care seeking behaviour in Bangladesh which is largely controlled by cultural beliefs such as religion and patriarchy [57], [59]–[61].

Further, a conjectural explanation for apparently higher incidence rate in males in the age groups 0–17, may be that young sons are more highly valued that young daughters and are therefore more likely to be taken for hospital treatment. The reversing of the situation in later years of life may more truly reflect the exposure of women to infection during acts of cleaning and caring.

The reason for there not being a single reported case in the age group of 5–9 both for male and female is currently unknown, but may be related to vaccination being provided to school-age children. In Bangladesh, the male population is more exposed for working and other purposes than females, which may explain the higher infection rates obtained for the males in the population [58]. Other factors such as greater mobility, social behavioral attitudes as mentioned above, and lack of immunity because of lack of previous exposure may also attribute to the disproportionate number of cases in the male population [16]. Further study is therefore needed to determine the underlying risk factors, to prevent certain gender and age groups being infected by typhoid fever.

The mean and median age of the cases was found to be 17 and 14 respectively, which contrasts earlier findings in Dhaka [8] however it is similar to the results of studies from Pakistan, Indonesia and India [18], [19], [23]. The present study further confirms that there is not significant variation in the occurrences of typhoid between urban and rural environment (urban, m: 5.72 SD: 6.34; rural, mean: 5.94, SD: 12.02, p>0.05). Since previous population-based studies have mainly been conducted in urban locations in South Asia, some bias may have occurred, implying that the disease is largely confined to urban areas [16]. Urban areas in South Asia are rapidly growing compared to other parts of the world, and often characterized by inadequate provision of safe water and sanitation, hence the burden of this disease seems to be higher in urban places than its rural counterpart. This may also be introduced due to the fact that urban populations can, and do, seek medical help more often than rural populations, which could affect the number of cases that are recorded in these two locations.

A distinct seasonal variation was found with almost half (45%) of the reported cases found to have occurred in the monsoon. This is contrasting to the finding of a prospective community-based study [4] but supports other results [18], [25]. Monthly distribution revealed that August to September had the highest cases while December to February showed relatively low cases. Environmental factors such as rainfall may have substantial influence to the occurrence of typhoid [12], [22] with increasing transmission of water borne pathogens during wet periods [62]. Because of heavy rainfall during the monsoon in South Asia, a peak of disease occurrence during July to October is not surprising as chances of surface water contamination is also high [18], particularly in densely populated areas like DMA. Although the case-fatality rate was relatively low during the study period, improvements to the water and sanitation infrastructures could reduce the risk of infection and fatality, hence reducing the disease burden.

The spatial association between water bodies and the incidences of typhoid showed significant relationships. This finding suggests that people living closer to water bodies may have elevated risk of infection. This relationship has not been reported earlier, however, case-control studies in India [18] and Vietnam [17] revealed that residents close to water bodies, and who use surface water for drinking tend to have more typhoid risk. A similar observation was also reported for diarrhoea incidence [56]. The areas supporting our hypothesis of inverse relationship between typhoid occurrence and distance to waterbodies might explained by the fact that there is a higher faecal contamination load in rivers [63]. As surface and groundwater water quality get severely degraded due to increasing anthropogenic activities in DMA, this may have significant impact on the transmission and distribution of typhoid. In addition, low income inhabitants in the study area frequently use surface water for cooking, bathing etc. As a result, contamination of these water bodies may have substantial impact on the disease dynamics in the communities. As S.Typhi bacteria can survive in water for days [64], contaminated surface water such as sewage, freshwater and groundwater would act as etiological agents of typhoid [65]. Inspection of the t-value and parameter estimate maps of typhoid infection and distance to water bodies further corroborates the spatial association of these two variables (Figure 4a&b). We found that mostly communities living close to the rivers Buriganga, Turag, and Balu had an elevated risk of typhoid infection compared with communities in other locations. These three rivers have been found to have extreme pollution loads throughout the year, measured in terms of coliform counts and other physio-chemical parameters [66]–[71], hence the assumption of an increase in the disease burden is warranted. Also, risk factor investigations for typhoid have shown that all source of drinking water, including pipe water, tube wells and surface water are perpetually highly contaminated in the study area [8], [25], and therefore increases the chance of water borne infection among people living in that area. The transmission dynamics of typhoid in relation to water quality, therefore remains a very promising area for further investigation. It is important to note that we have used major water bodies to regress against dependent variable which is in coarse resolution. Using a finer resolution water bodies map may provide further detail as people in the study area depend on small waterbodies such as ponds for their domestic and bathing purposes.

The global autocorrelation analysis using the Moran's *I* demonstrated that the spatial distribution of typhoid was clustered for all years (2005–2009) (Table 4), signifying that the disease is not uniformly or randomly distributed over DMA. This information can guide public health professionals in their search for possible interventions. An interesting distribution pattern was observed in the typhoid incidence map (Figure 5), namely, that typhoid infections reported in the *mahalla's* were often located close to water bodies such as river network, lakes and ponds. One may conclude from this distribution that people closer to water bodies are more likely to be affected by typhoid fever because of huge pollution loads of surface water bodies, and the spatial regression analysis carried out in this study also supports this finding.

The LISA map (Figure 6) indicated that significant spatial clustering of census tracts with regard to typhoid endemicity in DMA. Our result suggests that empirical Bayesian-smoothed typhoid rates were spatially dependent for the years 2005–2009. This study identified 3 multi-centred and five single-centred clusters. These spatial cluster maps can be used as an initial step in the development of disease risk prediction map since neighbouring spatial units tend to share similar environments and are often connected by the spread of communicable disease [72]. Typhoid incidences in the study area have been reported to be correlated with socio-economic, environmental and sanitation factors [8], [25]. Therefore, an integrated study considering socio-economic, environmental and other relevant factors would greatly benefit public health community in deeper understanding of the dynamics and transmission of typhoid risk in DMA or elsewhere. Since rapid urbanization and food habits tend to alter the prevalence of typhoid [2], this study underscore the necessity of the implementation of sustained safe water and sanitation associated with rapid urban expansion in DMA.

**Figure 8:**
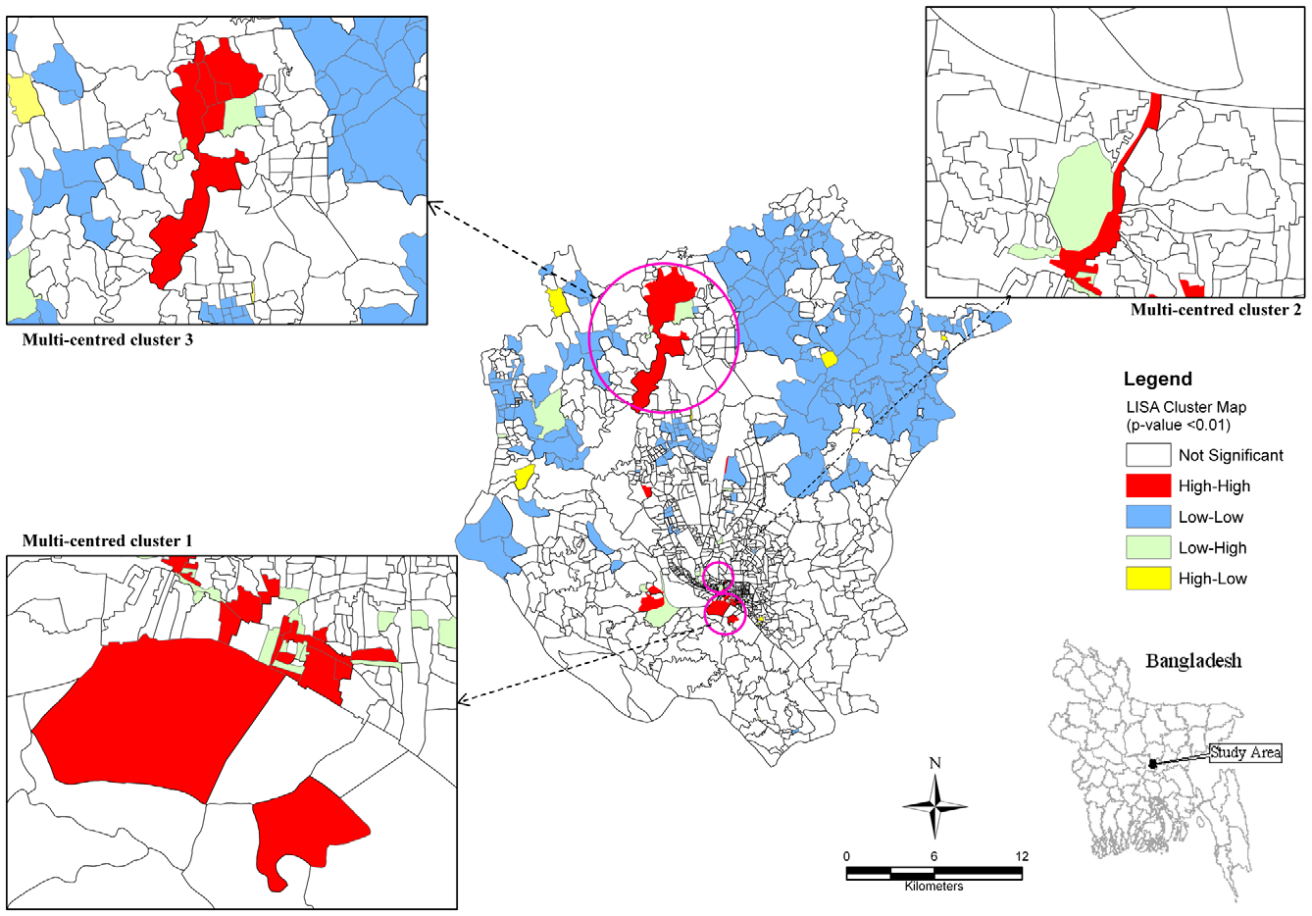
Spatial clusters (hotspots) of typhoid in DMA during 2005–2009. See Figure S3 for high resolution version.

The temporal analysis of the relationship between typhoid cases and hydro-meteorological factors revealed that the number of reported cases was amplified by increases in temperature, rainfall and river levels (Figure 8). While the seasonal distribution that we found in this study was similar to the distributions reported in earlier studies, one study by Lin et. al. [73] reported a contradictory finding for the association between river levels and typhoid incidences in Vietnam. Vapour pressure, temperature and precipitation have elsewhere been found to have significant associations with enteric diseases [12], [22], [74], which substantiates the result of this study. Our statistical model further stipulates that increase in rainfall and temperature lead to the higher typhoid cases in the study area. Since flooding is pervasive during the monsoon in DMA, increases in rainfall during the rainy season pollute the surface water which may have caused higher incidences of typhoid [75]. In addition, tube wells that are also flooded during the monsoon may be another source of infection due to contamination with faecal organisms [76], [77]. This study suggests that safe water supply remains a key issue in developing strategies for controlling typhoid infection in DMA.

Our study is not without limitations. First of all, the disease data that were acquired from hospitals may have underestimated or overestimated the typhoid records. Because the data were historical records and documented from the record room of each hospital, we had no valid method to ascertain repeated hospitalizations of an individual patient. In addition, hospital-based surveillance may underestimate actual infected population because only people in a severely weakened state tend to get admitted for treatment. Secondly, we only consider 11 major health service providers, the majority of which were public hospitals. The study could be improved by including data from private clinics where most of the affluent members of the population seek health services. Thirdly, we also could not separate cases into typhoid and paratyphoid groups. Isolation of these two types would allow us to estimate the disease dynamics and identify the most prevalent disease in DMA. Fourthly, the use of two or more methods to identify clustering is suggested as different analytical methods may recognize different underlying spatial patterns in the same dataset [78]. In this study, only one clustering method was used. Therefore, a future study should employ other spatial analytical technique to validate the result.

Despite the limitations above, the major strength of this study is the derivation of the first fine-scale regional map of the spatial distribution of typhoid and its epidemiology in Bangladesh.

## Conclusions

Using multi-temporal typhoid data and spatial analytical methods, this study explored the epidemiology and spatial patterns of typhoid infection in DMA of Bangladesh. Epidemiological characteristics showed that the disease disproportionately affects the male population and certain age groups. We did not notice any significance on the occurrence of typhoid between urban and rural areas. Seasonal analysis showed that the risk of typhoid infection is high during monsoon. Temporal distribution suggested that the disease is increasing with time which underscores the importance of prevention. Cluster maps that have developed in this study would help planners to assess spatial risk for typhoid incidences in DMA or elsewhere, and to derive appropriate health policy.

The findings of this study could contribute to the understanding of spatial variability of the burden of disease at the community level and may be useful in making decisions about vaccination. Local public health officials can use the information to identify the areas having higher disease occurrences and prepare for targeted interventions. For example, children can be targeted for immunization as other measures such as improvement of water supply and sanitation require what would be a huge investment for a resource-poor country. In this study, spatial and environmental factors were used to identify possible causal factor for typhoid incidences. In addition to these factors, other variables such as population density can be used to examine the factors that are most responsible at the local level. To prevent the spread of typhoid, awareness program should be initiated for the people who rely on nearby water bodies for drinking and domestic purposes. Because of recurrent flooding in the study area in the monsoon season, infected debris could have been another source of disease transmission that would increase the risk of acquiring the disease. Therefore, typhoid prevention can be addressed through both short- and long-term measures. As a short-term measure, people should be informed through a targeted campaign program of the dangers of using unboiled surface water during the monsoon. Medium-term measures could include the improvement of drainage facilities to minimize runoff of human waste into water bodies and long-term measures may be the development of a strong surveillance system to identify both cases and carriers. Finally, an efficient vaccination program can be undertaken for age-specific population at risk, though vaccines are not an alternative to safe water and good hygiene practices [79].

## Supporting Information

Figure S1 **Spatial regression between typhoid incidence (per 100,000 people) and distance to water bodies.** A) Shows spatial distribution of the t-value, B) shows the parameter estimates. High resolution version of Figure 5.
(TIF)

Figure S2 **Spatial variation in the occurrence of typhoid infection.** This shows the raw annual incidence rate(A) and EB-smoothed incidence rates (B) from 2005 to 2009 in census districts in DMA: High resolution version of Figure 6.
(TIF)

Figure S3 **Spatial clusters (hotspots) of typhoid in DMA during 2005–2009.**
(TIF)

Figure S4 **Sensitivity analysis.** Percent change (and 95% CIs) in the number of typhoid cases for (A) river level (per 0.1 m increase above the threshold), (B) rainfall (per 10 mm increase below threshold) and (C) temperature (per 1°C increase) with each number of harmonics and indicator variable of month (M). Presented results are from final models adjusted for seasonal variation (8 harmonics), inter-annual variations, and public holidays.
(TIF)

Checklist S1 STROBE checklist
(DOC)
