# Supplementary figures and images for: Typhoid Fever and Its Association with Environmental Factors in the Dhaka Metropolitan Area of Bangladesh: A Spatial and Time-Series Approach
Source: PLoS Negl Trop Dis. 2013 Jan 24;7(1):e1998. doi: 10.1371/journal.pntd.0001998 (PMC3554574; doi:10.1371/journal.pntd.0001998)

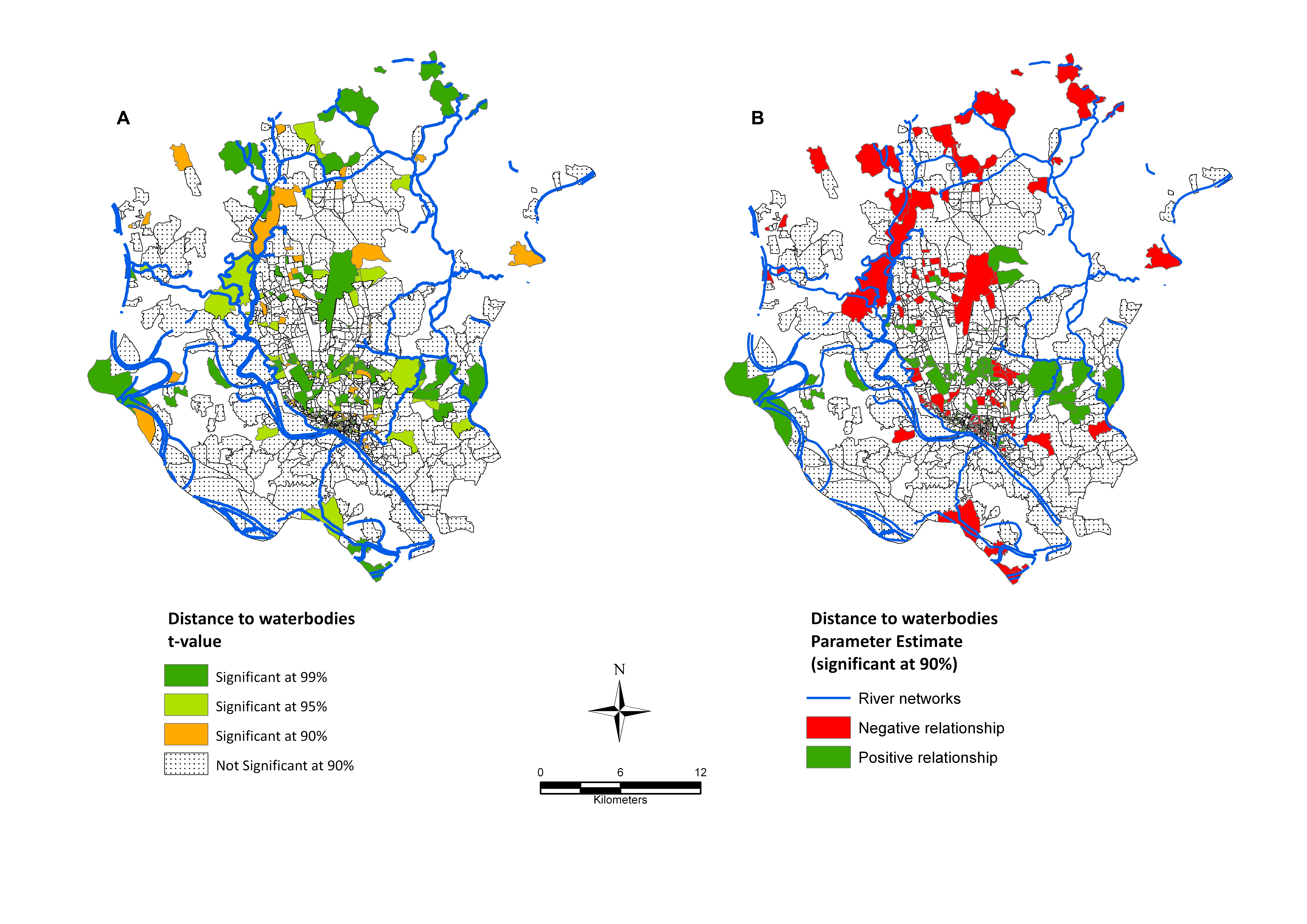

Supplement: Figure S1 — Spatial regression between typhoid incidence (per 100,000 people) and distance to water bodies. A) Shows spatial distribution of the t-value, B) shows the parameter estimates. High resolution version of Figure 5. (TIF) [file pntd.0001998.s001.tif]

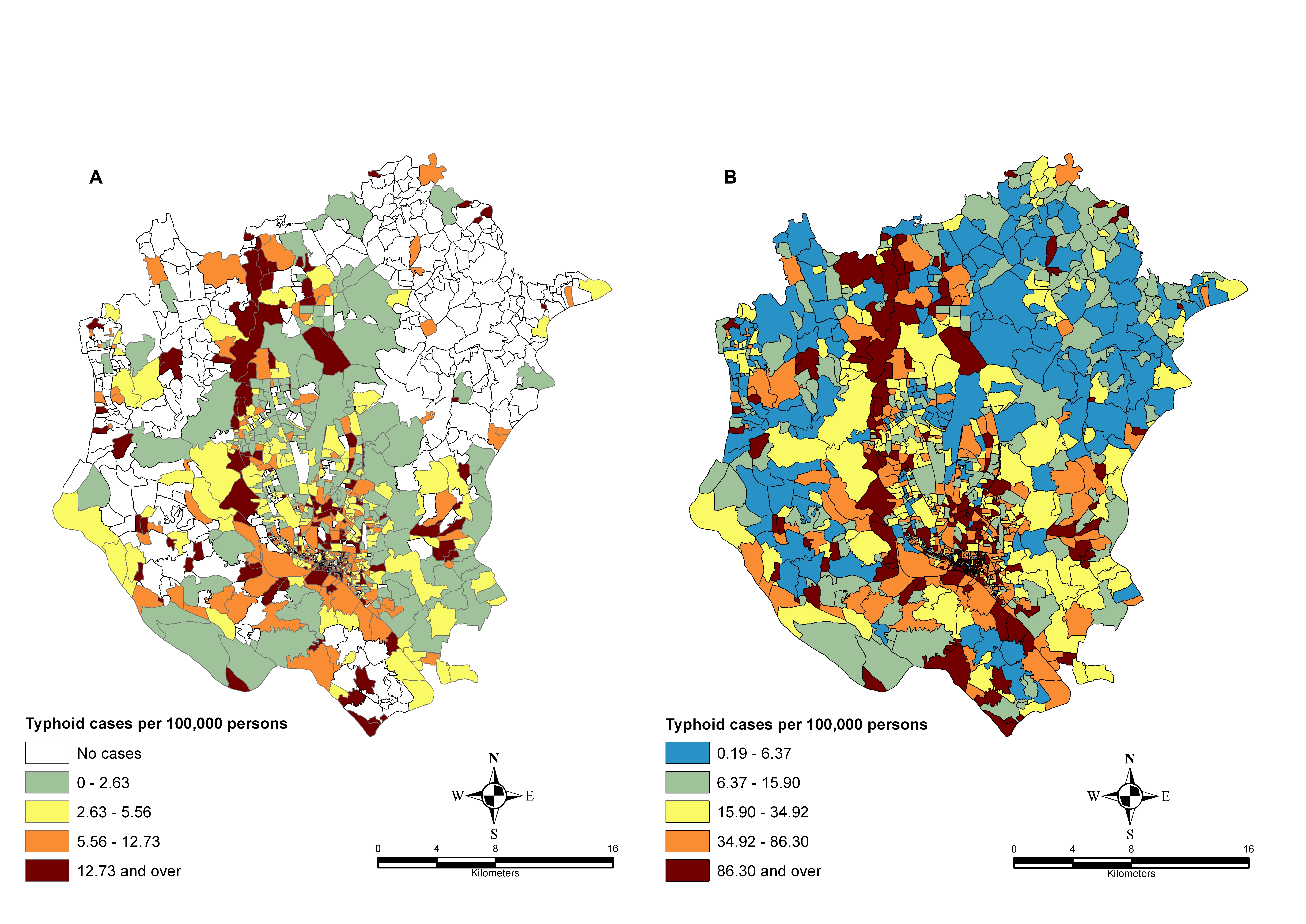

Supplement: Figure S2 — Spatial variation in the occurrence of typhoid infection. This shows the raw annual incidence rate(A) and EB-smoothed incidence rates (B) from 2005 to 2009 in census districts in DMA: High resolution version of Figure 6. (TIF) [file pntd.0001998.s002.tif]

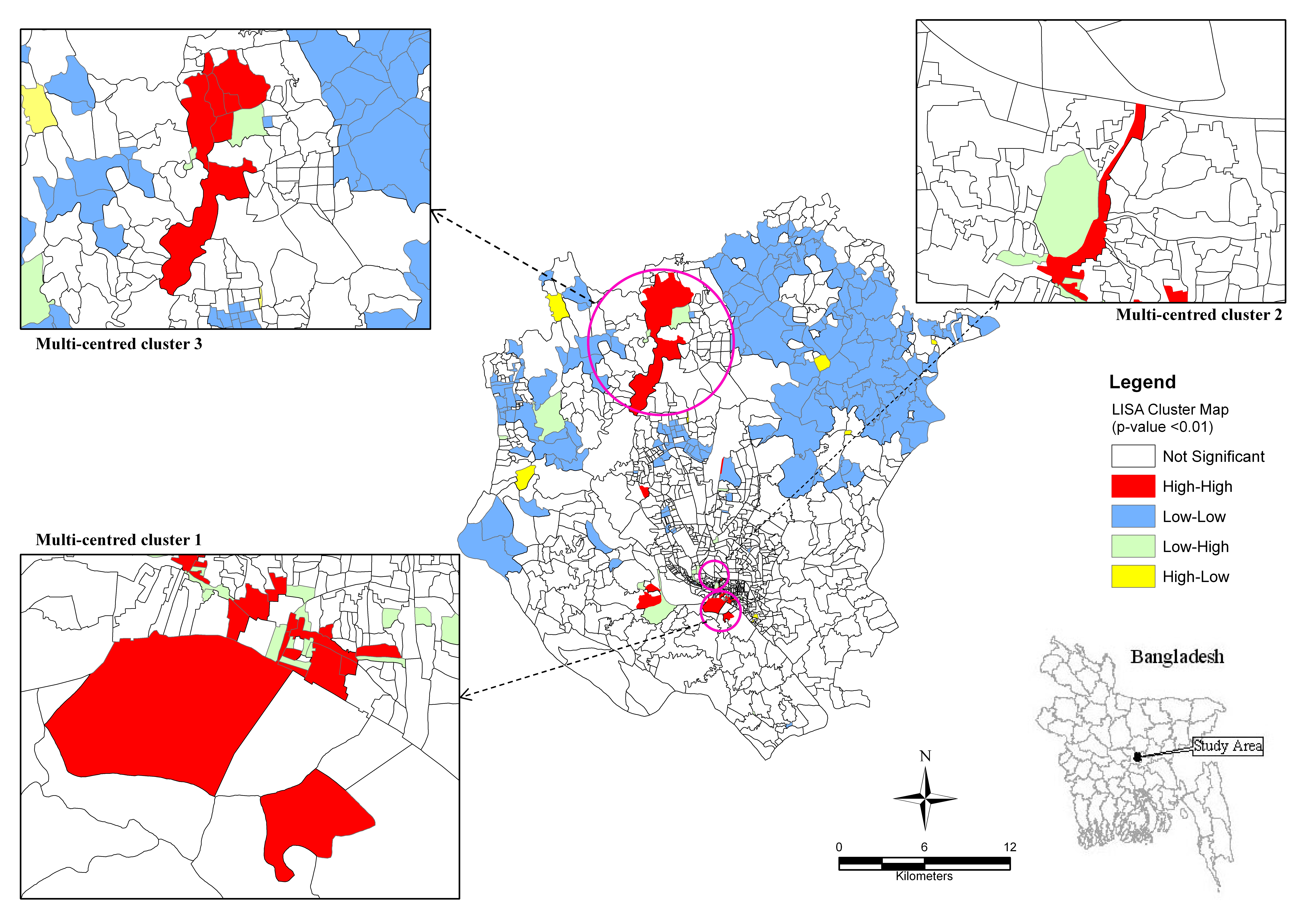

Supplement: Figure S3 — Spatial clusters (hotspots) of typhoid in DMA during 2005–2009. (TIF) [file pntd.0001998.s003.tif]

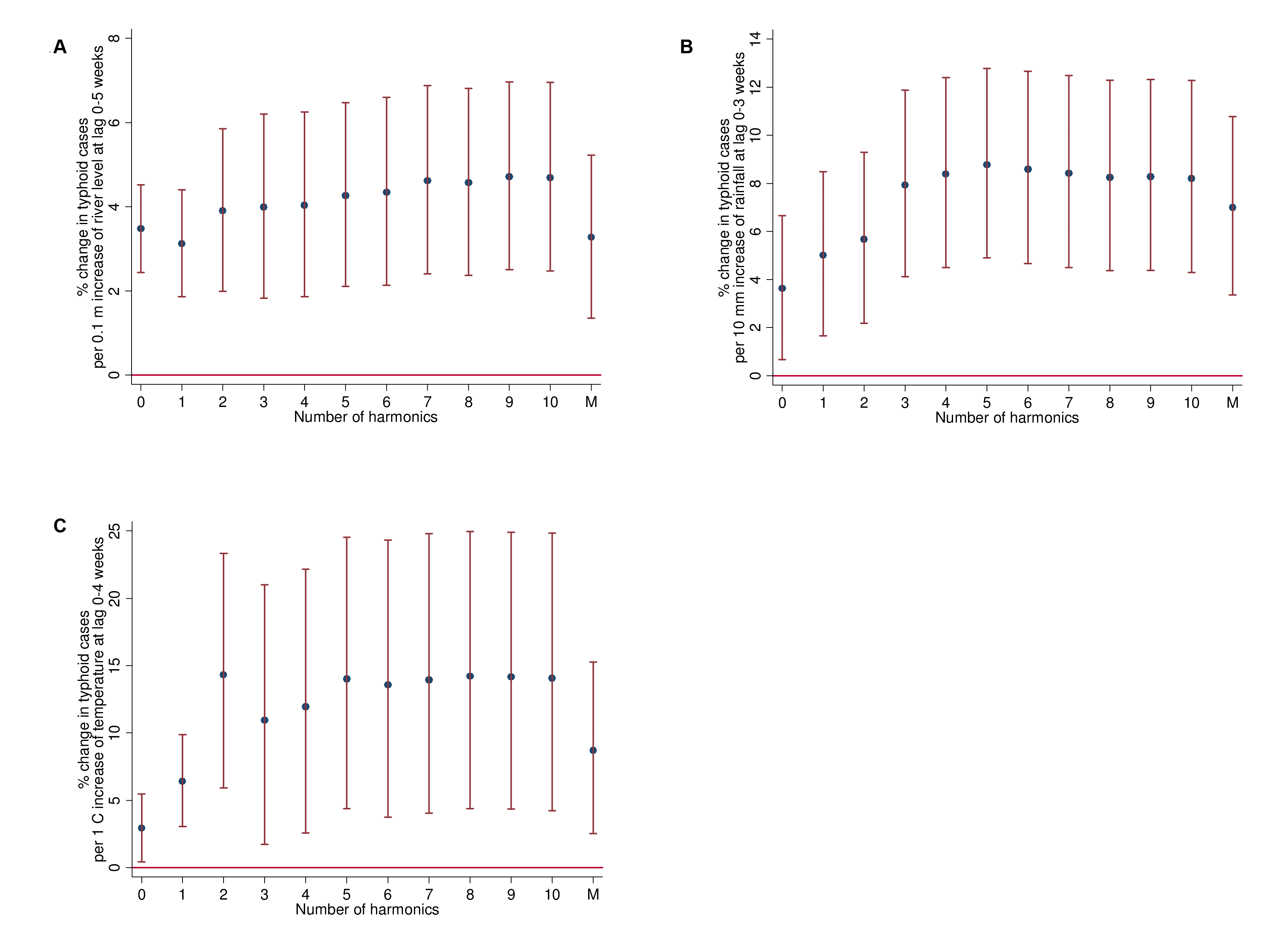

Supplement: Figure S4 — Sensitivity analysis. Percent change (and 95% CIs) in the number of typhoid cases for (A) river level (per 0.1 m increase above the threshold), (B) rainfall (per 10 mm increase below threshold) and (C) temperature (per 1°C increase) with each number of harmonics and indicator variable of month (M). Presented results are from final models adjusted for seasonal variation (8 harmonics), inter-annual variations, and public holidays. (TIF) [file pntd.0001998.s004.tif]
